# Dialysis

**Published:** 1862-10

**Authors:** Louis Elsbern


### DIALYSIS.

### A REPORT OF A NEW MEANS OF CHEMICAL ANALYSIS.

BY LOUIS ELSBERN, M. D.

Lecturer on "the application of remedies," in the University of N. Y.

(1.) Graham on Liquid Diffusion applied to Analysis. *Royal Society Translations*, London, 1861. Part I. *American Journal of Pharmacy*, 1861., p. 518.
(2.) Redwood on Dialysis, *London Pharmaceutical Journal*, April, 1862.
(3.) Daubeny on Agricultural Chemistry, *Gardeners' Chronicle*, London, December 7 14, 1862.
(4.) Procter on Dialysis, *American Journal of Pharmacy*, July, 1862.
(5.) Dialysis. Editorial Leader, *London Medical Times and Gazette*, Aug. 2. 1862.

THE art of chemical analysis is truly advancing with rapid strides. Accurate and untiring, enthusiastic yet laborious experimenters lead the van; and it requires the dedication of a life's mission promptly and thoroughly to follow. The medical practitioner, while his science makes it an imperative duty to familiarize himself with the stand-point which the great auxiliary and ally of his art has reached, finds it no easy task to keep himself *au courant* with even the methods of progress. Volumetrical and microscopical analyses have but just been made available for the purposes of the physician; and "while yet fascinated with the beauties and subtleties of spectral analysis, our attention is again claimed for another analytical discovery—less beautiful, it is true, and less subtle, but susceptible of much wider application, yielding results of greater practical value, and therefore possessing more immediate interest to us as medical men." (5.) The new method here alluded to—dialysis—we owe to the elaborate researches of Prof. Thomas Graham, the Master of the Mint, of London. It consists in effecting analysis by means of liquid diffusion. It requires only a most simple apparatus, dispenses almost entirely with chemical reagents, and is very easy to apply. Indeed, "it may fairly be described as a kind of royal road or short cut, enabling us to arrive at analytical results previously unattainable, or attainable only by processes far more complicated, and far more open to fallacy." (5.) I have thought it not unprofitable to transcribe from the sources indicated above—and especially from the last two, which themselves are reports based upon the preceding—a sketch of the nature of this new process, the mode of its practical application, and the valuable results that already have been and may yet be expected to be derived from it.

I have defined "dialysis" to be the method of effecting chemical analysis by means of liquid diffusion. Liquid diffusion has been defined as "the tendency which particles of matter in solution have to move from one part of a liquid menstruum to another, even in opposition to the force of gravity, under particular circumstances." (4.) It is well known that if a solution of common salt, for instance, be conveyed to the bottom of a jar of distilled water so carefully that the fluids be not mixed mechanically,—either by means of a pipette or by placing the saline solution in a smaller jar —the dissolved salt will be found, after standing some time undisturbed, to have diffused itself into the distilled water. Now it has been ascertained (1.) that the same substance in aqueous solution always diffuses itself into the same superincumbent medium at the same rate within a certain time; while "different substances in solution of equal strength diffuse unequally in equal times." (2.) Thus common salt diffuses into water at least twice as fast as Epsom salt, and the latter twice as fast as gum arabic. Of all the bodies hitherto tested (4.) hydrochloric acid is the most diffusive; assuming this body as unity, the time of equal diffusion for chloride of sodium is 2·33; for sugar 7; sulphate of magnesia, 7; albumen, 49; caramel, 98. Mr. Graham discovered that, as a general rule, crystallized bodies diffuse much more readily than amorphous substances; hence, he classed highly diffusible substances together as *Crystalloids*, and gave to the feebly diffusible ones the name of *Colloids*, from *collin*, gelatine, the type of this class. Among the colloids are hydrated silicic acid, hydrated albumina, and other soluble metallic peroxydes, isomorphous with the latter body, together with gelatine, albumen, starch, dextrin, and the gums, caramel, vegetable and animal extractive matters. (5.) Mr. Graham considers crystallizable bodies to be in a *statical* condition, while the colloids are in a *dynamical* state; that is to say, the latter have a greater susceptibility to the influence of external agents, and from this cause are adapted to the transforming influences of the processes of organic life, like albumen, gelatin, and fibrin of the animal body.

If, instead of a single substance, we use a mixed solution of two or more substances in our experiment with liquid diffusion, it is plain that what before was mere diffusion, now becomes a diffusive separation, for as the most diffusive body travels most rapidly, it will show itself first and most largely in the upper strata of superincumbent liquid. And separation of two or more substances will, of course, be the more complete the greater the difference between their respective diffusibilities. Furthermore, bodies of feeble diffusibility,—colloids—will permit a highly diffusible substance—a crystalloid—in aqueous solution in contact with them to diffuse or pass through or into them, while they will refuse a passage, partially or wholly, to a body of the colloid class. Taking advantage of this fact, Mr. Graham found on trial that a very thin stratum of a gelatinous medium would prevent the diffusion of colloid bodies, and that by the use of such a septum or diaphragm, the process of separating the crystalloids from the colloids becomes greatly simplified. We can now amplify our definition of dialysis, which "is nothing more than the diffusive separation of crystalloid from colloid bodies through a septum of gelatinous matter, the septum allowing the passage of the one and not of the other." (4 and 5.)

The whole apparatus needed is a "dialyser' and a basin. The dialyser consists of a membranous septum stretched across a hoop or band of gutta percha six to ten inches in diameter and two inches high, and tied with a string, so as to form a vessel like a drum-head or tambourine. The basin is a dish or larger glass vessel of similar shape about six inches deep.

For use, the basin is three-fourths filled with distilled water, the smaller vessel is floated on it, and the liquid to be diffused poured into the latter, when the process proceeds without further attention.

By placing the liquid to be diffused at the top instead of at the bottom of the water, the process is much facilitated, especially where the diffusible body is abundant. The solution of the matter which passes the septum is called the diffusate. Parchment-paper, "made by simply passing ordinary white wrapping-paper quickly through a cool mixture of two parts of sulphuric acid and one of water, by weight, and then washing it thoroughly till the acid is removed," (4.) has been found to be the best septum, although paper starched with starch jelly, or gelatin, or coagulated albumen, and others, would answer. Redwood says (2.): "It may be well to observe that parchment paper, or indeed any of the substances named above, can only be used as a septum in dialysing *aque-*

*ous* liquids. The septum is considered by Mr. Graham to owe its action to its condition, in the wetted state, as the hydrate of a colloidal substance, in which the water of hydration is held by a weak affinity, which the superior affinity exerted by a crystalloid can overcome. Hence there is dehydration taking place in one direction, and rehydration in the other direction, through the septum during its action. A septum suitable for dialysing alcoholic and ethereal solutions remains to be discovered. Some form of collcdion may possibly answer the purpose."

Mr. Graham found that half a litre of urine, dialysed for twenty-four hours, gave its crystalloidal constituents to the external water. The latter, on evaporation, yielded a white saline mass, from which urea was extracted by alcohol in so pure a condition as to appear in crystalline tufts upon the evaporation of the alcohol.

As to the practical application and uses of dialysis, we find enumerated—

1st. It permits of the isolation of various chemical substances in a state of purity in which we were not previously aware of their being able to exist. For instance, chemists had hitherto never succeeded in obtaining a perfectly pure solution of silica. The solution obtained by treating silicate of soda with chlorohydric acid was not pure, always containing a certain quantity of the acid and of chloride of sodium, which resisted all further attempts at separation. But by subjecting the solution to the process of dialysis, the acid and salt, being crystalloids, diffuse out, while the silica, being a colloid, remains behind dissolved in water, and perfectly pure. In like manner, dialysis enables us to obtain solutions of peroxide of iron, alumina, and several other bodies, perfectly free from the salts or other chemical agents hitherto indispensable to their solution. (5.)

2d. It affords a means of separating crystallizable and well defined bodies from amorphous bodies, like extractive, gums, mucilages, the pectin-like substances, the tannins, oils, etc. For instance, if a vegetable extract containing an alkaloid or crystalline neutral bitter principle, be rubbed down with water to a uniform mixture and thrown on the dialyser, this active principle, together with all the outside earthy matter, will be found in the diffusate, while the inert ingredients will be retained. Mr. John Attfield (*Pharm. Journal, March*, 1862, p. 447) has applied this apparatus in an investigation of the mineral constituents of plants, especially as exhibited in the saline efflorescences on the vegetable extracts so familiar to all apothecaries. Mr. Attfield also suggests its use for purifying crude lemon juice.

Mr. Graham has separated the crystallized ingredients of urine. Baron Liebig has isolated creatin from extract of meat; and more recently he has demonstrated by its means the presence of *alloxan* in an animal secretion. (*Pharm. Jour.*, 528, *April*, 1862.)

Mr. Buchner has shown (ibid. 572) that when a thick viscous mucilage of marshmallow root is put into the dialyser, over distilled water, after two days the whole of the asparagin of the root is in the first diffusive, and may be obtained in fine crystals by simple evaporation. (4.)

3d. It may become of the greatest use in "the separation of the more active crystallizable constituents of vegetable substances from inert colloidal matter, and *the production in this way of a new class of medicines*, containing the more active principles of plants, partially purified, and in the state of combination in which they exist in nature." (2.) Such preparations would occupy an intermediate place between tinctures, decoctions, and extracts, on the one hand, and the pure active principles which they often contain (such as alkaloids) on the other. The advantages of vegetable remedies in this form would be greater uniformity of strength, certainty of action, and convenience of administration. They would also keep better, and being void of all inert matter, they would be *purely* medicinal. The main difficulty in the way of their preparation is the very dilute condition of the solution, and the slowness of the operation, and consequent tendency to spoil and ferment; but it may prove, owing to ferments being colloidal matter, that the diffusates may not be liable to change to a degree that will embarrass the operation. Prof. Redwood is now engaged in applying dialysis to opium and aloes.* (4 and 5.)

4th. In medico-legal inquiries, it affords a most valuable means of separating arsenious acid and the various poisonous metallic salts, as well as vegetable poisons, such as strychnia, morphia, and the other poisonous alkaloids, from the crude

*In the forthcoming work on New Remedies, by Drs. Perry and Elsberg, due prominence will be given to this new class of medicines, based on original investigations, by dialysis, of many, especially indigenous articles colloidal contents of the stomach and intestines and other organic solutions. For instance, let a portion of tissue suspected to contain arsenic be chopped into small pieces, soaked in pure water, and then thrown on the dialyser. At the end of twenty four hours or so, the arsenic, even if its quantity be infinitesimally small, will have been diffused into the external water in a state fit for the immediate application of chemical tests. The poison is thus eliminated, free from all organic impurity, and without employing any agent at all liable to contain it—an advantage which any one will not fail to appreciate who is conversant with the usual process. Mr. Redwood has "already obtained these substances by dialysis from the stomach, the flesh and the blood of animals that have been poisoned, distilled water alone being used for their extraction." (2.)

5th. It affords a partial explanation of certain points in physiology and in the knowledge of the modus operandi of medicines which have hitherto been involved in much obscurity. As to the physiology of digestion, the mucous membrane of the stomach and intestines may be compared to a dialytic septum between the blood on the one side, and the blood-making constituents of food on the other. Dilute liquids taken into the stomach diffuse through (or as we generally say, are absorbed by) its mucous membrane. The plastic constituents of food, on the other hand, being colloids, "are retained in the stomach, while the act of digestion proceeds under the influence of crystalloids that are dialysed into that organ, and then pass on to undergo new changes connected with absorption, assimilation, and excretion." (2.) Also, "the action of medicines must be considerably influenced by the state in which they exist as crystalloids and colloids. Thus, iron in the state of chloride, sulphate, or other crystalloidal salt, would be diffused through the walls of the stomach; but not so if in the state of a colloid, such as basic chloride or basic nitrate, in which state it would pass into the intestines, exerting its action probably through the entire length of the alimentary canal." (2.) When we know more of the comparative diffusive power of different medicated preparations than we do at present, we shall probably prescribe them with greater success. (5.)

6th. The transmission of sap through a plant, the separation of its various secretions from each other, and their maintenance in a state of isolation in appropriate receptacles, are phenomena also in part explained by the principle of dialysis. Although the sap is propelled upwards through the plant partly by capillary attraction, partly by atmospheric pressure, owing to the evaporation from the leaves and the partial vacuum thereby produced—it makes its way into the plant, in the first instance, by endosmosis through the spongiodes of the roots. The peculiar juices of plants—starch, gum, etc., —are generally colloids, and therefore have no tendency to pass through the walls of the cells in which they have been elaborated; the different acid and alkaline products, on the other hand, being crystalloids, permeate membrane freely, "but are only temporary constituents or steps in the series of changes which are intended to convert carbonic acid into sugar and starch, and they are consequently got rid of either by exosmosis or else by some chemical process by which they are converted into glocose or fruit sugar." (3.)

Lastly, the principle of dialysis has likewise important bearings on the nature of the ultimate molecules of matter, and on certain geological phenomena. It is believed that its further investigation will perhaps throw considerable light on molecular action as a source of electricity, heat, and other imponderable influences, and lead to some knowledge of the essence or real condition of matter.—*Am. Med. Times.*

